# Cardiac rehabilitation in patients who underwent primary percutaneous coronary intervention for acute myocardial infarction: determinants of programme participation and completion

**DOI:** 10.1007/s12471-017-1039-3

**Published:** 2017-09-15

**Authors:** M. Sunamura, N. ter Hoeve, M. L. Geleijnse, R. V. Steenaard, H. J. G. van den Berg-Emons, H. Boersma, R. T. van Domburg

**Affiliations:** 1 Capri Cardiac Rehabilitation Rotterdam, Rotterdam, The Netherlands; 2 Department of Rehabilitation Medicine, Erasmus MC, Rotterdam, The Netherlands; 3 Department of Cardiology, Thorax Center Erasmus MC, Rotterdam, The Netherlands; 4 Cardiovascular Research School COEUR, Erasmus MC, Rotterdam, The Netherlands

**Keywords:** Percutaneous coronary intervention, Cardiac rehabilitation, Participation rates, Completion rates

## Abstract

**Background:**

Hospital length of stay after acute myocardial infarction (AMI) treated with primary percutaneous coronary intervention (pPCI) has reduced, resulting in more limited patient education during admission. Therefore, systematic participation in cardiac rehabilitation (CR) has become more essential. We aimed to identify patient-related factors that are associated with participation in and completion of a CR programme.

**Methods:**

We identified 3,871 consecutive AMI patients who underwent pPCI between 2003 and 2011. These patients were linked to the database of Capri CR, which provides dedicated, multi-disciplinary CR. ‘Participation’ was defined as registration at Capri CR within 6 months after pPCI. CR was ‘complete’ if a patient undertook the final exercise test.

**Results:**

In total, 1,497 patients (39%) were registered at Capri CR. Factors independently associated with CR participation included age (<50 vs. >70 year: odds ratio (OR) 7.0, 95% confidence interval (CI) 5.1–9.6), gender (men vs. women: OR 1.9, 95% CI 1.3–1.8), index diagnosis (ST-elevation myocardial infarction [STEMI] vs. non-ST-elevation myocardial infarction [NSTEMI]: OR 2.4, 95% CI 2.0–2.7) and socio-economic status (high vs. low: OR 2.0, 95% CI 1.6–2.5). The model based on these factors discriminated well (c-index 0.75). CR programme completion was 80% and was inversely related with diabetes, current smoking and previous MI. The discrimination of the model based on these factors was poor (c-index 0.59).

**Conclusions:**

Only a minority of AMI/pPCI patients participated in a CR programme. Completion rates, however, were better. Increased physician and patient awareness of the benefits of CR are still needed, with focus on the elderly, women and patients with low socio-economic status.

## Background

Standard care for patients with an acute myocardial infarction (AMI) consists of immediate primary coronary percutaneous intervention (pPCI) [1]. Usually, patients with an uncomplicated AMI are then referred to a non-pPCI hospital for further care within a few hours, and discharged home within 2 to 4 days. Although a short hospital length of stay implies a lesser burden on the patient, it does result in more limited time for patient education. Therefore, participation in a cardiac rehabilitation (CR) programme is essential for AMI patients [2].

CR is a class I recommended intervention in coronary artery disease (CAD) patients [3, 4] with beneficial effects on physical fitness, quality of life, cardiovascular risk factors, and cardiovascular mortality and morbidity [5]. Nevertheless, merely one third of CAD patients are referred to CR in the Netherlands [6]. Better understanding of referral and participation patterns is essential to improve optimal utilisation of CR. We therefore aim to identify patient-related characteristics that are predictive of CR participation and completion in AMI patients treated with pPCI.

## Methods

### Study population and data collection

We identified all AMI patients who underwent pPCI in the Erasmus Medical Center Rotterdam between 2003 and 2011. These patients were linked to the database of Capri CR, which provides dedicated CR for patients who undergo pPCI in the Erasmus MC.

Data on cardiac risk factors, clinical patient characteristics and treatment were prospectively collected in a database as part of the ongoing pPCI registry at the Erasmus MC. Capri CR provided information on participation and completion of the CR programme.

This study was approved by the Erasmus MC Ethics Committee (MEC-2009-080).

### Cardiac rehabilitation

Capri CR provides standardised outpatient CR according to the European Society of Cardiology (ESC) guidelines on CR [2]. The multi-disciplinary programme focuses on improving physical condition, self-confidence and social integration. The programme consists of 1.5-hour group exercise sessions twice a week during a maximum of 12 weeks, plus courses on how to deal with exercise, diet, smoking cessation and stress management. The aim is to improve adherence to lifestyle modification and help patients to adopt a positive role in the care of their own health. The exact length of a CR programme is determined by a multidisciplinary team together with the patient, with a minimum of 6 weeks.

‘Participation’ was defined as registration at Capri CR within 6 months after pPCI. CR ‘completion’ was defined as at least 75% attendance at the physical programme, based on the methodology described by Beauchamp et al. [7].

### Statistical analysis

Normality of continuous variables was not rejected by Shapiro-Wilk tests. Hence, continuous variables are presented as mean and standard deviation. Categorical variables are summarised as numbers and percentages. Differences in characteristics between patients with and without CR participation, and with and without CR completion were evaluated by Student’s t‑tests (continuous variables), and Chi-square or Fisher’s exact tests (categorical variables).

Univariate and multivariate logistic regression analyses were applied to investigate which baseline characteristics were related with CR participation or CR completion. We considered age, gender, body mass index, cardiac history (prior MI, prior coronary artery bypass graft, prior PCI), diabetes, hypertension, dyslipidaemia, smoking, family history, socio-economic status and disease presentation as potential explanatory variables. Variables that reached statistical significance in univariate analysis entered the multivariate stage. Socio-economic status was based on the patient’s postal code. We applied the 4‑category classification developed by the Netherlands Institute for Social Research, which accounts for the average income in the corresponding city district, the percentage of people with a low income, the percentage of people with low-level education and the percentage of people without a paid job. Regression analysis results are reported as odds ratios (OR) and 95% confidence intervals (CI). We used the C‑index to assess the discriminatory ability of the multivariate models.

All statistical tests were two-tailed and p‑values were considered statistically significant at *p* < 0.05. Analyses were performed using IBM SPSS Statistics version 21.

## Results

During 2003–2011, 4,260 AMI patients underwent pPCI in the Erasmus MC. A total of 352 died within 60 days, whereas another 37 were lost to follow-up. The remaining 3,871 patients were eligible for analysis.

### Capri CR participation

**Fig. 1:**
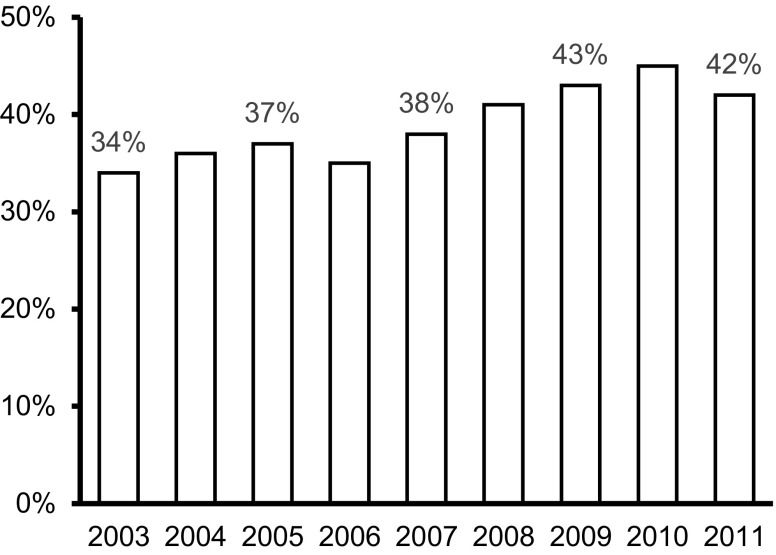
Participation rate over the years. Percentages of patients after primary percutaneous coronary intervention for acute myocardial infarction who participated in cardiac rehabilitation

The number of patients participating in the Capri CR programme amounted to 1,497 (39%). This percentage remained fairly consistent during the 8‑year study period (Fig. 1) with a tendency to improvement. Capri CR participants were younger, had a better socio-economic status and a more favourable CAD risk profile (except smoking) than non-participants (Tab. 1). Participants were less often female. While 27% of the AMI patients were women, this percentage was lower (20%) in the CR group than in the non-CR group (32%). Furthermore, participants less often had a history of cardiovascular disease. Age, socio-economic status and diagnosis were independently associated with Capri CR participation (Tab. 2). Patients below the age of 50 years had a 6.9 times higher chance of participation than patients aged 70^+^. The chance of CR participation was 2.0 times higher in patients who belonged to the upper social-economic class (as compared with the lowest class), and 2.4 times higher in those presenting with ST-elevation myocardial infarction (STEMI). The c‑index of the multivariate model that predicted Capri CR participation based on these three characteristics was 0.75, implying a fair discriminatory performance.

**Table 1:** Clinical and socio-economic characteristics of the study population according to participation in and completion of the CAPRI cardiac rehabilitation program

	Participation	No participation	*P*-value	Completion	No completion	*P*-value
Number of patients	1497	2374		1193	304	
*Age, years*	56.9 (10.3)	64.5 (12.4)	<0.001	57.0 (10)	56.4 (11)	0.38
*Age in categories*						0.26
<50	400 (27%)	285 (12%)	<0.001	308 (26%)	92 (30%)	
50–60	800 (53%)	937 (40%)		652 (55%)	148 (49%)	
60–70	120 (8%)	280 (12%)		96 (8%)	24 (8%)	
>70	177 (12%)	866 (36%)		137 (11%)	98 (13%)	
Men	1198 (80%)	1614 (68%)	<0.001	963 (81%)	235 (77%)	0.2
*Socio-economic status*			<0.001			<0.005
Lower class	702 (47%)	1358 (57%)		543 (46%)	159 (52%)	
Lower middle class	344 (23%)	535 (23%)		273 (23%)	71 (23%)	
Upper middle class	184 (12%)	246 (10%)		149 (12%)	35 (11%)	
Upper class	265 (18%)	232 (10%)		226 (19%)	39 (13%)	
*Diabetes*	173 (12%)	513 (22%)	<0.001	123 (10%)	50 (16%)	<0.005
*Hypertension*	594 (40%)	1193 (50%)	<0.001	473 (40%)	121 (40%)	1.00
*Dyslipidaemia*	629 (42%)	1306 (55%)	<0.001	500 (42%)	129 (42%)	0.87
*Current smoking*	614 (41%)	719 (30%)	<0.001	465 (39%)	149 (49%)	<0.001
*BMI*			0.007			0.65
<18.5	4 (0%)	15 (1%)		3 (1%)	1 (1%)	
18.5–25.0	344 (23%)	625 (26%)		277 (23%)	67 (22%)	
25.0–30.0	928 (62%)	1348 (57%)		739 (62%)	189 (62%)	
>30	219 (15%)	383 (16%)		172 (14%)	47 (15%)	
*Prior MI*	220 (15%)	689 (29%)	<0.001	161 (13%)	59 (19%)	<0.001
*Prior CABG*	30 (2%)	236 (10%)	<0.001	23 (2%)	7 (2%)	0.70
*Prior PCI*	158 (11%)	600 (25%)	<0.001	116 (10%)	42 (14%)	0.04
*Presentation with STEMI*	1070 (71%)	1072 (45%)	<0.001	862 (72%)	208 (68%)	

Continuous data are presented as mean (standard deviation)

Categorical data are presented as numbers (%)

*BMI* body mass index, *MI* myocardial infarction, *CABG* coronary artery bypass graft, *PCI* percutaneous coronary intervention, *STEMI* ST-elevation myocardial infarction

**Table 2:** Predictors of participation in the CAPRI cardiac rehabilitation program

	Univariate analysis		Multivariate analysis	
	OR	95% CI	OR	95% CI
*Age, years*				
<50	6.9	5.50–8.57	7.0	5.06–9.57
50–60	4.2	3.46–5.04	3.6	2.90–4.55
60–70	2.1	1.60–2.74	2.3	1.67–3.17
>70	1		1	
*Men*	1.9	1.62–2.20	1.5	1.26–1.77
*Socio-economic status*				
Lower class				
Lower middle class	1.2	1.06–1.46	1.3	1.09–1.57
Upper middle class	1.4	1.17–1.79	1.3	1.01–1.60
Upper class	2.2	1.81–2.69	2.0	1.60–2.48
*Diabetes*	0.47	0.39–0.57		
*Hypertension*	0.65	0.57–0.74		
*Dyslipidaemia*	0.59	0.52–0.67		
*Current smoking*	1.6	1.40–1.83		
*BMI*				
<18.5	0.48	0.16–1.47		
18.5–25.0	1			
25.0–30.0	1.3	1.07–1.46		
>30	1.0	0.84–1.28		
*Prior MI*	0.42	0.36–0.50		
*Prior CABG*	0.18	0.13–0.27		
*Prior PCI*	0.35	0.29–0.42		
*Presentation with STEMI*	3.0	2.65–3.49	2.4	2.03–2.77

*OR* odds ratio,* CI* confidence interval*, BMI* body mass index*, MI* myocardial infarction, *CABG* coronary artery bypass graft, *PCI* percutaneous coronary intervention, *STEMI* ST-elevation myocardial infarction

### Capri CR completion

Altogether 1,193 (80%) participants completed their CR programme. Programme completion was associated with socio-economic status, and inversely associated with CAD risk factors and CAD history (Tab. 1). In multivariate analysis, diabetes, current smoking and a history of MI were inversely related with the odds of CR programme completion (Tab. 3). However, the multivariate model that aimed to predict CR completion had poor discriminatory performance (c-index 0.59).

**Table 3:** Predictors of completion of the CAPRI cardiac rehabilitation program

	Univariate analysis		Multivariate analysis	
	OR	95% CI	OR	95% CI
*Age, years*				
<50	0.98	(0.64–1.49)		
50–60	1.28	(0.87–1.92)		
60–70	1.16	(0.66–2.08)		
>70	1			
*Men*	1.23	(0.35–1.96)		
*Socio-economic status*				
Lower class	1			
Lower middle class	1.12	(0.82–1.54)		
Upper middle class	1.25	(0.83–1.89)		
Upper class	1.69	(1.16–2.50)		
*Diabetes*	0.59	(0.41–0.83)	0.59	(0.40–0.88)
*Hypertension*	1.00	(0.77–1.28)		
*Dyslipidaemia*	1.00	(0.76–1.25)		
*Current smoking*	0.67	(0.52–0.85)	0.59	(0.46–0.78)
*BMI*				
<18.5	0.71	(0.75–7.14)		
18.5–25.0	0.91	(0.69–1.28)		
25.0–30.0	0.91	(0.58–1.35)		
>30	1			
*Prior MI*	0.67	(0.52–0.85)	0.63	(0.40–0.84)
*Prior CABG*	0.83	(0.35–1.96)		
*Prior PCI*	0.67	(0.46–0.98)		
*Presentation with STEMI*	1.20	(0.92–1.59)		

*OR* odds ratio,* CI* confidence interval*, BMI* body mass index*, MI* myocardial infarction, *CABG* coronary artery bypass graft, *PCI* percutaneous coronary intervention, *STEMI* ST-elevation myocardial infarction

## Discussion

Only two out of five AMI patients who underwent pPCI during 2003–2011 in the Erasmus MC participated in the Capri CR programme. Apparently, patients and physicians did not adhere to the ESC guidelines recommendations for long-term management after CAD [3, 8]. Patient’s adherence to CR fails to a larger extent. Particularly, elderly patients, female patients, patients presenting without ST-elevation and patients with lower socio-economic status were underrepresented among CR participants. Once started, an encouraging four out of five patients appeared able to complete Capri CR. Nevertheless, there is room for improvement, since non-completion was frequent in patients who could have benefited the most: diabetics, smokers and those with a past MI.

The observed low participation rate of 39% is consistent with earlier studies. It is even estimated that, on average, less than 30% of all eligible patients attend CR [9]. This may be especially worrying in patients after pPCI, when there is little time for patient education due to the short hospital stay. The ESC guidelines provide a Class I recommendation for ‘exercise-based rehabilitation’, with level of evidence B. The ESC guidelines for non-ST-elevation myocardial infarction (NSTEMI) have a Class IIa recommendation for ‘participation in a well-structured cardiac rehabilitation programme’. Indeed, it is underscored that ‘the benefits were established in the era preceding modern treatment of STEMI’, whereas ‘… in patients with an uncomplicated course, rehabilitation can often be performed on an outpatient basis with an efficacy similar to that of centre-based cardiac rehabilitation’ [8]. We believe that these and similar judgements may not reduce reluctance among treating physicians to refer to CR, whereas dedicated CR programmes have scientifically demonstrated positive effects on patient well-being and prognosis, also in the ‘modern era’ [10].

In literature, the terms ‘referral’ and ‘participation’ are often incorrectly used in the same context. If a cardiologist refers a patient to CR, but the patient is not willing to participate, this is incorrectly counted as ‘no referral’. We believe that ‘participation’ is the correct term in our study.

The low participation rate in elderly is a consistent finding, perhaps due to a lower expected benefit of CR for older patients [11]*.* Furthermore, older patients are more likely to have orthopaedic, vascular or neurological comorbidities which could prohibit or limit CR participation. It is a challenge for CR programmes to find ways to facilitate these kind of patients: sometimes by offering an individualised rehabilitation programme. Whether this is as effective as standard CR, has yet to be studied.

In our study, and in several other studies, women were also less likely to participate [12]. However, this is not a consistent finding in the literature [13]. Women in our study were older, had a higher prevalence of cardiac risk factors and less often STEMI. All factors that were all predictive of lower participation to CR. Still female gender was independently associated with low participation to CR. The reasons why women are less likely to participate in CR or other cardiac interventions are yet still poorly understood [14].

Patients with a lower socio-economic status were less likely to participate. In American studies, it is often reported that the low participation rate is caused by insurance problems [15]. In the Netherlands, however, CR is fully reimbursed in the compulsory basic health insurance with a participation rate of >99%. Nevertheless, in our study higher socio-economic status was still associated with higher CR participation. This may be due to a lack of understanding of the benefits of CR and/or logistical problems in patients with a lower socio-economic status [16]. It has been demonstrated that if logistical problems are solved by providing a tailored CR programme for this specific group of patients, outcomes may be different [6, 15, 17].

Based on the patient-related data we collected, CR completion could be poorly predicted. Our somewhat discouraging results regarding CR completion by diabetic patients are consistent with a recent study by Armstrong et al. [18]. Interestingly, they found that diabetics who completed CR had a significant mortality reduction. This emphasises the importance for diabetics to complete CR despite their complexity and higher incidences of co-morbidities, potentially precluding completion of the physical part of the CR programme. Also, it cannot be excluded that diabetics already have so many contacts with health care providers that they are physically or mentally not able to continue the twice weekly training sessions.

Non-completion of patients with a prior MI could be explained by former CR participation. However, similarly to diabetes, patients with a prior MI have adverse prognosis, which could be caused by impaired left ventricular function. That condition may hamper participation in CR. Although, like Forman et al. state, CR programmes see opportunities for this category of patients by starting home-based programmes using latest technologies [19]. The future will tell whether this is the solution.

A review by Gaalema et al. described identical findings as in our study regarding smokers, namely a higher participation rate but also a higher rate of premature quitting [20]. Why smokers do more frequently quit CR remains an unresolved issue.

Several interventions to stimulate participation and completion of CR have been studied. Reviews suggest that approaches aimed at motivating patients may be improving CR participation, for example invitation calls or visits early after discharge, followed by the use of self-management techniques [21, 22]. The 2014 Cochrane Database Systematic Review by Karmali et al. confirms these positive results of motivational calls and visits to increase participation [23]. To stimulate completion there were some positive but biased results on supervised or unsupervised exercise, accompanied by a variety of self-management techniques [24, 25].

The authors conclude that there is still not enough evidence to make practice recommendations for increasing participation and completion of CR. Particularly, studies to identify useful interventions to stimulate under-representing patient groups such as women and elderly are still missing. We hypothesise that individually tailored approaches may increase the likelihood of success. Clark et al. conclude more or less the same by mentioning participation in CR as a consumer behaviour, in which interventions influencing family support, patient-friendly scheduling, and other socially and individually related factors can have a positive role [26].

## Limitations

It should be noted that our study is observational, retrospective, and based on a single-centre experience (Erasmus MC/Capri CR). Some factors that might be related to participation in and not completion of CR may be missing in our study. For example, the influence of distance and transportation options to the CR location was not incorporated in our analysis. Patient socio-economic status was not based on individual data, but on area of residence, which is only a proxy for socio-economic status. In addition, physician’s endorsement of the benefits of CR was not analysed in our study. We did not find written records in patient files stating that the patient had indeed been referred. The fact that younger age, male gender, STEMI and higher socio-economic status were predictive of participation to CR suggest that cardiologists have the idea that these patients most likely benefit from CR. The use of an automatic referral system may aid in increasing referral rates by helping to disregard personal feelings of the referring physician [27, 28].

Although not completing a CR programme might be related to poor outcome, it should be emphasised that the duration of CR should always be tailored to the individual patient. At one end of the spectrum, a short CR period of six weeks for a patient who is already physically active and participating at work may suffice, whereas improvements in physical and mental health may require more than the traditional 12 weeks of CR in the socially vulnerable patient [29].

## Conclusion

Participation in cardiac rehabilitation after pPCI for AMI was poor. Even with better completion rates, only a minority of total AMI patients completed a CR programme. Patients who are elderly, female or of low socio-economic status appear to be particularly at risk of CR non-participation and non-completion. Therefore, these patient groups should be targeted in order to enhance their participation and completion of CR.
